# The Effect of Resuscitation Residents on the Duration of Pre-induction of Targeted Temperature Management in Out-of-Hospital Cardiac Arrest

**DOI:** 10.7759/cureus.32050

**Published:** 2022-11-30

**Authors:** Patrick Wloszczynski, David A Berger, David M Lee, Nai-Wei Chen, Michael J Burla

**Affiliations:** 1 Emergency Medicine, Beaumont Hospital, Royal Oak, USA; 2 Internal Medicine, Loyola University Medical Center, Maywood, USA; 3 Research, Beaumont Hospital, Royal Oak, USA; 4 Emergency Medicine, Southern Maine Health Care, Biddeford, USA

**Keywords:** pre-induction, emergency medicine, cardiac resuscitation, out-of-hospital cardiac arrest, targeted temperature management

## Abstract

Background

The Resuscitation Rotation is a novel second-year emergency medicine rotation focusing on the highest acuity patients, including out-of-hospital cardiac arrest (OHCA). The resuscitation resident (RR) functions as an extra physician during resuscitation and post return of spontaneous circulation (ROSC). The objective of this study is to examine if the presence of a RR decreases the pre-induction interval of targeted temperature management (TTM) for patients following OHCA.

Methods

A retrospective study was conducted at a tertiary care level 1 trauma center with an annual ED census of 127,323 visits in 2019. We retrospectively reviewed consecutive OHCA patients from September 1, 2014, to July 20, 2020, who underwent TTM. Patients were identified as cases with or without a RR. Clinical characteristics were summarized by the status of RR involvement and compared by using t-test and χ^2^ test for continuous and categorical variables, respectively. All tests with p < 0.05 were considered to indicate statistical significance.

Results

Our study population identified 198 adult OHCA patients that underwent TTM from 2014-2020. There were exclusions for missing TTM start time and for missing patient characteristics leaving 176 for final analysis, of which 55 (33.3%) had RR involvement. The mean time (hours) to TTM initiation (ie, the pre-induction phase) for patients involving the RR versus those without was not statistically significant (3.11 vs 3.34, p=0.39). Linear regression analysis indicates that the adjusted effect of RR involvement was not associated with the mean hours of pre-induction (p=0.47).

Conclusion

There is no statistically significant association of a RR on the duration of the pre-induction phase. Limitations include that both arms had prolonged pre-induction phases. This may represent a non-optimized TTM protocol. Future work will aim to use the RR to improve our pre-induction phase.

## Introduction

In the United States, 347,322 adults sustain emergency medical service (EMS)-assessed non-traumatic out-of-hospital cardiac arrest (OHCA) annually [1,2]. Based on national data from the Cardiac Arrest Registry to Enhance Survival (CARES) database of 2013-2018, adult non-traumatic OHCA overall survival to discharge and survival with favorable neurologic outcome (as gauged via a cerebral performance category (CPC) 1/2) is 10.4% and 8.2%, respectively [2]. The outcomes from the State of Michigan over this same duration in CARES have lagged behind at 8.5% and 7.0%, respectively.

There is substantial variation in overall survival and CPC-1/2 survival across Michigan hospitals [3]. Survivors may experience significant morbidities such as poor neurologic outcomes and prolonged disability [4]. With this in mind, methods to improve neurologic outcomes following the return of spontaneous circulation (ROSC) play a significant role in fortifying the chain of survival [5].

Targeted temperature management (TTM) has demonstrated improved neurologic outcomes in patients following cardiac arrest [6,7]. Neurologic injury following cardiac arrest occurs rapidly and delays in cooling have been shown to negate the benefits of hypothermia [8,9]. Therefore, it is important for cooling to begin expeditiously and for hospitals to have communication and leadership that have been associated with improved survival [10]. Prior work at our institution did reveal significant variation in the use of TTM within the state of Michigan [11]. A potential method to facilitate the initiation of TTM is to have additional staff who are specifically designated to resuscitate critically ill patients.

Within our institution, a novel one-month rotation was developed that occurs during the second year of the emergency medicine residency [12]. During this rotation, a resident is assigned to the high acuity area of the emergency department (ED), and serves as an additional provider above normal staffing levels to assist in the management of critically ill patients. We theorize that the presence of an additional physician assisting in the care of these patients can result in improved processes. The objective of this study is to examine if the presence of a resuscitation resident (RR) decreases the pre-induction interval for patients following OHCA.

## Materials and methods

Study overview

A retrospective study was conducted at a tertiary care level 1 trauma center, Beaumont Hospital, Royal Oak, Michigan, United States, with an annual ED census of 127,323 visits in 2019. We retrospectively reviewed consecutive patients from September 1, 2014, to July 20, 2020. The inclusion criteria for our study were patients ≥ 18 years old admitted following OHCA with ROSC, who underwent TTM. Exclusion criteria included patients that were transferred from outside facilities after ROSC had been achieved as a RR had no possibility of being involved in their initial care. Additionally, patients were excluded if the time of initiation of TTM was not recorded.

Course description

As briefly illustrated in the introduction, the resuscitation rotation was developed as a part of the second-year curriculum. The RR spends one month in the ED focusing on high acuity patients, including cardiac arrest, sepsis, pulmonary embolism, cerebrovascular emergencies, trauma, and other acutely ill patients. the RR is scheduled to be in the high acuity pod of the department for 40 hours a week, and the attending on shift at that time supervises the care provided [12]. There is always a RR every month, and they are given specific education at the start of the rotation on goals of resuscitation, both with ROSC patients and other critically ill patients.

Study design

With the approval of the Beaumont Health Institutional Review Board (IRB number: 2018-093), data were abstracted from a prospectively collected database for the years September 1, 2014, to July 20, 2020. September 2014 was chosen as the start time to coincide with the inception of the resuscitation rotation. Encounters were collected from our institution’s electronic medical record system (Epic Systems Corporation, Verona, Wisconsin, United States). All RR encounters were recorded in a protected, web-based data bank, with a personal log-in (New Innovations, Uniontown, Ohio, United States). Patients were identified as cases with or without a RR.

Variables

The main independent variable of this study was the presence of RR. Covariates were work shift of residents (ie, a surrogate for the time ROSC occurred; coded as day-shift and night-shift) and patient characteristics including age, sex, race, BMI, initial core temperature, arrest witness status, cardiopulmonary resuscitation (CPR) bystander status, and rhythm type.

Outcome measure

The primary outcome of this study was the time to initiation of TTM (ie, pre-induction phase).

Data analysis

Continuous data were shown as means (standard deviations, SD) and categorical data as frequencies (percentages). Clinical characteristics were summarized by status of RR involvement and compared by using t-test and χ2 test for continuous and categorical variables, respectively.

We initially explored the influence of RR involvement and work shift on the duration of the pre-induction phase via a two-way analysis variance (ANOVA). We tested the interaction term of RR involvement and work shift and the result indicated that the interaction was not statistically significant (ie, the effect of RR involvement on duration of the pre-induction phase did not depend on work shift). Subsequently, in multivariable linear regression analyses, we fitted models without including the interaction term of RR involvement and work shift to assess the effect of RR involvement adjusted for work shift and other clinical patient characteristics with no potential issue of collinearity. All tests with p < 0.05 were considered to indicate statistical significance. All statistical analyses were performed with SAS software version 9.4 (2013; SAS Institute Inc., Cary, North Carolina, United States).

## Results

A total of 198 adult OHCA patients underwent TTM from 2014-2020. Fifteen were excluded for missing TTM start time and seven for missing patient characteristics, leaving 176 for the final analysis of which 55 (33.3%) had RR involvement. Patient characteristics are illustrated in Table 1. Overall, the mean age of patients was 61 (SD 16.1) with 64.8% males. There were more encounters during the day shift with both RR and non-RR involvement with patients with shockable rhythms.

**Table 1:** Patient-related and resident-related characteristics stratified by resuscitation-resident training BMI, body mass index; CPR, cardiopulmonary resuscitation. § For continuous variables, means ± standard deviations were presented. For categorical variables, frequencies and percentages were presented.

Variables	All	Resuscitation Resident (Yes)	Resuscitation Resident (No)	p-value
n	176	55 (31.3%)	121 (68.7%)	N/A
Resident characteristics				
Work shift				
Night shift	62 (35.2%)	13 (23.6%)	49 (40.5%)	0.03
Day shift	114 (64.8%)	42 (76.4%)	72 (59.5%)	
Patient characteristics				
Age, years	61.0 ± 16.1	61.7 ± 16.8	60.7 ± 15.8	0.70
Sex				
Male	114 (64.8%)	33 (60.0%)	81 (66.9%)	0.37
Female	62 (35.2%)	22 (40.0%)	40 (33.1%)	
Race				
White	132 (75.0%)	41 (74.5%)	91 (75.2%)	0.93
Non-White	44 (25.0%)	14 (25.5%)	30 (24.8%)	
BMI, kg/m^2^	30.2 ± 8.0	29.3 ± 7.2	30.6 ± 8.3	0.32
Initial core temperature, ℃	35.8 ± 1.5	35.9 ± 1.2	35.7 ± 1.6	0.48
Arrest witness status				
Unwitnessed	36 (20.4%)	7 (12.7%)	29 (24.0%)	0.09
Bystander	140 (79.6%)	48 (87.3%)	92 (76.0%)	
CPR status				
Non-bystander	84 (47.7%)	23 (41.8%)	61 (50.4%)	0.29
Bystander	92 (52.3%)	33 (58.2%)	60 (49.6%)	
Rhythm type				
Non-shockable	111 (63.1%)	42 (76.4%)	69 (57.0%)	0.01
Shockable	65 (36.9%)	13 (23.6%)	52 (43.0%)	

Table 2 shows that the mean time (hours) to TTM initiation (i.e., the pre-induction phase) for patients with RR involvement versus those without was not statistically significant (3.11 vs 3.34, p=0.39). Results further indicated that the impact of RR involvement on the pre-induction interval did not depend on the work shift (i.e., a surrogate for the time ROSC occurred). For example, the mean hours of pre-induction during day shift (3.48 vs 3.58, p=0.76) or night shift (2.54 vs 3.13, p=0.25) were not statistically significant. However, there was a statistically significant difference in the mean hours of pre-induction between day shift and night shift overall (3.51 vs 2.94, p=0.03).

**Table 2:** Initiation of TTM for mean time of pre-induction phase, hours, by resuscitation-resident training and work shift TTM, targeted temperature management

	Resuscitation Resident (Yes)	Resuscitation Resident (No)	Overall	p-value
Day-shift	3.48	3.58	3.51	0.76
Night-shift	2.54	3.13	2.94	0.25
Overall	3.11	3.34	N/A	0.39

Linear regression analyses examining the effect of RR involvement on the duration of the pre-induction phase are displayed in Table 3. Model 1 only includes non-patient characteristics (RR and work shift) and RR is not associated with a statistically significant decrease in mean hours of pre-induction (p=0.39) whereas work shift (day shift) was positively associated with mean hours of pre-induction (0.56; 95%CI 0.05 to 1.09, p=0.03). Model 2 adjusted for patient characteristics (age, sex, BMI, CPR status, and rhythm type) indicates that the adjusted effect of RR involvement was not significantly negatively associated (DB1) with the mean hours of pre-induction (p=0.47); however, the adjusted effect of work shift (day shift) demonstrates increased mean hours of pre-induction (0.55; 95%CI -0.00 to 1.09, p=0.05). No additional patient characteristics were found to have a statistically significant association with the pre-induction interval.

**Table 3:** Effects of resuscitation-resident training on initiation of TTM for time of pre-induction phase in ordinary linear regression analysis TTM, targeted temperature management; BMI, body mass index; CPR, cardiopulmonary resuscitation; β, regression coefficients; CI, confidence interval § Model 1 only included resident-related characteristics. ‡ Adjusted effects of resident-related characteristics were estimated in Model 2, controlling for patient characteristics.

	Model 1^§^		Model 2^‡^	
Variables	β (95% CI)	p-value	β (95% CI)	p-value
Intercept	3.06 (2.63 to 3.49)	< 0.001	2.94 (1.52 to 4.37)	< 0.001
Resuscitation Resident				
No	1 (Reference)	N/A	1 (Reference)	N/A
Yes	-0.23 (-0.77 to 0.30)	0.39	-0.21 (-0.77 to 0.35)	0.47
Work shift				
Night shift	1 (Reference)	N/A	1 (Reference)	N/A
Day shift	0.56 (0.05 to 1.09)	0.03	0.55 (-0.00 to 1.09)	0.05
Age, years	N/A	N/A	-0.003 (-0.02 to 0.01)	0.71
Sex				
Male	N/A	N/A	1 (Reference)	N/A
Female	N/A	N/A	0.09 (-0.45 to 0.63)	0.73
BMI, kg/m^2^	N/A	N/A	0.01 (-0.03 to 0.04)	0.73
CPR status				
Non-bystander	N/A	N/A	1 (Reference)	N/A
Bystander	N/A	N/A	0.10 (-0.41 to 0.60)	0.70
Rhythm type				
Non-shockable	N/A	N/A	1 (Reference)	N/A
Shockable	N/A	N/A	0.13 (-0.41 to 0.68)	0.63

## Discussion

Within our ED, the advent of the resuscitation rotation has provided residents with additional educational opportunities to be involved with critically ill patients [12]. In our study, we sought to assess the impact of the presence of a RR in OHCA patients with ROSC that received TTM. Our results demonstrated that there is no statistically significant association of a RR on the duration of the pre-induction phase and the effect of RR involvement did not depend on the work shift of residents. While the RR does have specific education at the beginning of the rotation on how to approach post-ROSC care, an increase in literature review over the duration of the rotation may be of benefit. However, we speculate that the lack in change of pre-induction phase duration was due to multiple confounding variables. Specifically, lack of nursing staff, logistics of initiated TTM, and education to ancillary staff on the importance of improving times in the pre-induction phase. Aside from further improvement on the resuscitation rotation, we believe that this can be improved with a more coordinated approach to caring for post-ROSC patients by having formal education for all stakeholders involved in care (i.e., nursing, techs, etc).

Post-ROSC care with TTM demonstrates a complex task with a heterogenous patient population that requires close care in the ED and often interdepartmental coordination with critical care, cardiology, and neurology physicians. While having an additional resident physician beyond usual staffing levels did not improve duration times on the pre-induction phase of TTM for OHCA patients, the authors believe there is an opportunity to improve. Prior work done assessing RR impact has reported improvement in initiating TTM in OHCA [13]. The RR has also demonstrated an impact in care not related to OHCA, such as improved compliance on sepsis bundle orders and facilitating care of septic shock patients [14],

A faster pre-induction stage of TTM has been shown to improve neurologic outcomes in post-ROSC patients [9,15]. Therefore, it is important for EDs to develop and refine protocols to ensure the prompt initiation of TTM. Large variability exists in not only the etiology of patients’ cardiac arrests but also post-arrest management. The concept of “high-quality TTM” is now working its way into hospitals, as committees are formed for the sole purpose of integration and compliance with TTM protocols. Wyse et al. demonstrated an overall decrease in mortality following their TTM protocol implementation [16]. Within our hospital, a multi-disciplinary committee has been formed with the intention to implement an improved TTM protocol with the primary goal of shortening the stage of the pre-induction phase. Our RR serves as an invaluable opportunity to aid in this effort.

Strategies to optimize pre-induction and induction continue to be actively investigated. The impact of pre-hospital cooling and intra-arrest cooling has been investigated. Following ROSC coupled with in-hospital cooling, pre-hospital cooling has been shown to be an effective method of decreasing pre-hospital temperature and is associated with favorable neurologic outcomes at hospital discharge [17]. Bernard et al. reported their randomized controlled trial (RCT) where pre-hospital cooling was associated with a shorter time to target temperature without demonstrating a change in favorable neurological outcomes [18]. This study was an RCT that only assessed non-shockable rhythms, and did not include patients with shockable rhythms.

With regard to induction, studies have suggested that a faster time to target temperature may actually be associated with worse patient outcomes [19]. Some patients that achieve target temperature quickly have been found to have hypoxic damage to the thermoregulatory centers of the brain. As a result, they are unable to resist TTM resulting in a faster time to the target temperature. As more information continues to be produced with regard to the various stages of TTM, our understanding and our ability to treat our patients will continue to grow.

Limitations

This study has several limitations including its being retrospective and the small sample size. Additionally, RRs were only present for 31.3% of all this TTM cohort making the two arms unbalanced. This discrepancy is due to the fact that the RR is not present in the ED at all times. Within the resident arm, there may be inter-resident discrepancies between their management style and involvement within the case. For example, those that are farther along in their second year may possess a better knowledge of inter-departmental protocols of patients undergoing TTM resulting in expedited initiation. Furthermore, due to widespread inter-hospital variation in terms of TTM protocols, this study may not be generalizable when compared to hospitals that utilize different target temperatures, care for dissimilar patient populations, and apply different TTM inclusion/exclusion criteria. Finally, within this study, both arms had prolonged pre-induction phases. This may represent a non-optimized TTM protocol on which no RR would be able to have a significant impact.

## Conclusions

There is no statistically significant association of a RR on the duration of the pre-induction phase when initiating TTM on ROSC patients. Effect of RR involvement did not depend on work shift of residents. There may be other benefits of the RR being involved in the management of ROSC patients that were not specifically assessed in this study. Future work will aim to use the RR to improve our pre-induction phase and other aspects of ROSC patient care.
